# Role of hypoxia inducible factor 1α/Bcl-2/adenovirus E1B 19-kDa interacting protein 3 in alleviating effect of interleukin-4 on cerebral ischemia reperfusion injury in mice

**DOI:** 10.4314/ahs.v22i3.39

**Published:** 2022-09

**Authors:** Tianjing Wang, Chang Liu

**Affiliations:** 1 Department of Geriatrics, Daqing Oilfield General Hospital, Daqing 163001, Heilongjiang Province, China; 2 Department of Neurology, Daqing Oilfield General Hospital, Daqing 163001, Heilongjiang Province, China

**Keywords:** Hypoxia inducible factor 1α, Bcl-2/adenovirus E1B 19-kDa interacting protein 3, interleukin-4, cerebral ischemia reperfusion injury

## Abstract

**Background:**

Cerebral ischemia reperfusion injury (CIRI) is the pathophysiological basis of various cerebrovascular diseases. The aim of this study was to explore the role of HIF-1α/BNIP3 in the alleviating effect of IL-4 on CIRI in mice.

**Methodology:**

Mice were randomly divided into sham operation (Sham), ischemia reperfusion (IR), IL-4, HIF-1α inhibitor 2ME2 and IL-4+2ME2 groups. Middle cerebral artery occlusion model was established. After 24-h reperfusion, neurologic deficit score (NDS) was given. Cerebral infarction volume and brain water content were measured by 2,3,5-triphenyltetrazolium chloride staining and dry-wet weights, respectively. Apoptosis was detected by TUNEL staining. SOD, MDA and ROS levels, and HIF-1α, BNIP3, LC3II and Beclin-1 expressions were detected through colorimetry and Western blotting, respectively.

**Results:**

Compared with IR group, NDS, cerebral infarction volume, brain water content, apoptosis rate, and MDA and ROS levels decreased, while SOD, HIF-1α, BNIP3, LC3-II and Beclin-1 levels increased in IL-4 group (P<0.05). 2ME2 and IL-4+2ME2 groups had decreased NDS, cerebral infarction volume, brain water content, apoptosis rate and MDA, ROS, HIF-1α, BNIP3, LC3-II and Beclin-1 levels, but increased SOD level compared with those of IL-4 group (P<0.05).

**Conclusion:**

IL-4 reduces apoptosis and oxidative stress through activating the HIF-1α/BNIP3 pathway, thereby alleviating mouse CIRI.

## Introduction

Cerebral ischemia reperfusion injury (CIRI), defined as the worsened tissue and functional damage caused by the recovery of blood perfusion after cerebral ischemia, is the pathophysiological basis of a variety of cerebrovascular diseases. CIRI has a complex mechanism, and it has been found that oxidative stress, inflammatory response, apoptosis and autophagy are all important players therein^1^. Among them, autophagy can not only repair neuronal damage through eliminating damaged or redundant organelles, but also regulate neuronal death through affecting apoptosis and necrosis^2^. As an important part of CIRI, the inflammatory cascade reaction may play a key role^3^. Leukocytes release considerable pro-inflammatory mediators, and different inflammatory factors are released uncontrollably through the cascade reaction, thereby aggravating local inflammatory response and ultimately leading to destruction of the blood-brain barrier, cerebral edema, neuronal necrosis and hemorrhagic transformation^4^. Interleukin-4 (IL-4) is an important anti-inflammatory factor able to increase the secretion of anti-inflammatory factors such as IL-10 and TGF-β and inhibit the release of inflammatory factors such as IL-1 and TNF-α, exerting an anti-inflammatory effect. It has been confirmed that IL-4 can enhance autophagy, ameliorate CIRI in mice and improve long-term cognitive function, protecting the brain^5^. Hypoxia inducible factor 1α (HIF-1α) is an important transcription regulator in cells under hypoxic conditions, which is restrained under normoxic conditions. Bcl-2/adenovirus E1B 19-kDa interacting protein 3 (BNIP3) is a downstream target gene of HIF-1α. There is a close correlation between autophagy and hypoxia, and hypoxia-induced autophagy has a protective effect on cells. After activation of the HIF-1α/BNIP3 signaling pathway, increased autophagy is induced, alleviating myocardial IRI (MIRI). Therefore, this study intends to explore the role of HIF-1α/BNIP3 signaling pathway in the effect of IL-4 of alleviating CIRI in mice, thereby providing references for further explanation of its mechanism.

## Methods

### Laboratory animals

A total of 60 Balb/c mice of SPF grade (half male and half female, 2 months old, 20–25 g) were purchased from Haowei Biotech Co., Ltd. (SYXK (Tianjin) 2019-0006). They were fed in separate cages for 1 week under 23–25°C, standard humidity of 55–60% and 12/12 h day-night cycle, and had normal drinking and eating.

### Main reagents and apparatus

The following reagents and apparatus were used: recombinant mouse IL-4 (PeproTech, USA), rat anti-mouse IL-4 neutralizing antibody (Verax, USA), 2ME2 (Selleck, USA), HIF-1α, BNIP3, microtubule-associated protein 1 light chain 3-II (LC3II) and Beclin-1 antibodies (CST, USA), horseradish peroxidase-labeled secondary antibodies (Beijing Bersee Science and Technology Co., Ltd., China), 2,3,5-triphenyltetrazolium chloride (TTC) staining kits (Sigma, USA), terminal deoxynucleotidyl transferase-mediated dUTP nick end labeling (TUNEL) staining kits (Roche, USA), bicinchoninic acid (BCA) protein concentration assay kits (Beijing Zhongshan Goldenbridge Biotechnology Co., Ltd., China), superoxide dismutase (SOD), malondialdehyde (MDA) and reactive oxygen species (ROS) assay kits (Nanjing Jiancheng Bioengineering Institute, China), Thermo microplate reader (Shanghai Thermo Instrument Co., Ltd., China), refrigerated centrifuge (Beckman, USA), protein electrophoresis instrument (Bio-Rad, USA), and H7500 transmission electron microscope (Hitachi, Japan).

### Animal modeling and grouping

All of the 60 mice were divided into sham operation group (Sham group), ischemia reperfusion group (IR group), IL-4 group, HIF-1α inhibitor 2ME2 group (2ME2 group) and IL-4+HIF-1α inhibitor 2ME2 group (IL-4+2ME2 group) using a random number table. The middle cerebral artery occlusion (MCAO) model was established by thread technique in IR group, IL-4 group, 2ME2 group and IL-4+2ME2 group. Specifically, after anesthesia by intraperitoneal injection of 3% pentobarbital sodium, the mice were fixed on the operating table in a supine position. After hair shaving and disinfection, the left internal carotid artery was carefully exposed and inserted with a nylon thread until there was slight resistance at the distal end, and the thread was fixed. After ischemia for 60 min, the thread was withdrawn, followed by 24-h reperfusion in the ischemic region to establish the MCAO model. After operation, penicillin (200,000 U) was injected intramuscularly for 3 consecutive days. In Sham group, no MCAO was performed, and the remainng operations were the same as above. In IL-4 group, 2ME2 group and IL-4+2ME2 group, the corresponding IL-4 complex solution (10 µg of recombinant mouse IL-4 antibody and 50 µg of recombinant rat anti-mouse IL-4 antibody were mixed and diluted to 50 µg/mL with normal saline containing 1% mouse serum) and 2ME2 solution (15 mg/kg) were injected intraperitoneally and via the caudal vein at 30 min before modeling. After 24-h reperfusion, the neurologic deficit score (NDS) was given, and then the mice were sacrificed, from which brain tissues were harvested.

### NDS evaluation

NDS was given according to the Longa's method and the neurologic deficit symptoms were recorded at 14 d after modeling. The scoring criteria were as follows: 0 points: There are no neurologic deficit symptoms; 1 point: The left forelimb cannot be fully stretched; 2 points: The body turns to the left while walking; 3 points: The body leans to the left while walking; 4 points: paralyzed, unable to stand.

### Detection of volume of cerebral infarction by TTC staining

After anesthesia by 3% pentobarbital sodium, the mice were decapitated and their brains were harvested. The brain tissues were frozen in a refrigerator at -80°C for 5 min and prepared into 2 mm-thick coronal sections. Then the sections were incubated with 2% TTC solution at room temperature for 30 min (normal tissues were stained red, while infarction tissues were stained white), fixed in 4% paraformaldehyde solution overnight, and photographed equidistantly with a digital camera. The image was imported into a computer, and the volume of cerebral infarction was calculated using Image-Pro Plus6.0.

### Measurement of brain water content by dry-wet weight method

Part of the brain tissues (weight A) were placed in tinfoil and weighed (weight B). Then they were placed in an oven, dried and repeatedly weighed till the constant weight C. Wet weight =B-A, and dry weight =C-A. Finally, brain water content was calculated: (wet weight of brain tissues - dry weight of brain tissues)/wet weight × 100%, that is, (B-C)/(B-A) × 100%.

### Detection of apoptosis by TUNEL staining

Part of the brain tissues were prepared into paraffin sections, routinely deparaffinized, dehydrated with gradient ethanol, incubated with 3% H^2^O^2^ at room temperature for 10 min, digested with proteinase K at 37°C for 10 min, and incubated with TUNEL mixture away from light at 37°C for 1 h, followed by dehydration with gradient ethanol and immersion in xylene. Finally, the sections were sealed with neutral balsam, and the staining results were observed and photographed in 5 randomly-selected fields of view under an optical microscope (400×).

### Determination of SOD, MDA and ROS levels by colorimetry

Part of the brain tissues were washed with normal saline, prepared into homogenate with 10% of tissues and centrifuged. Then the supernatant was harvested, and the levels of SOD, MDA and ROS werdetected according to the instructions of kits.

### Determination of expressions of HIF-1α, BNIP3, LC3II and Beclin-1 by Western blotting

One portion of brain tissues was homogenized with cell lysis buffer to extract the total protein, and the protein concentration in each group was determined using BCA kits. After quantification and denaturation, the protein was separated by SDS-PAGE, quickly transferred onto a PVDF membrane, blocked with TBST solution containing 5% skim milk at room temperature for 2 h, and incubated with the HIF-1α, BNIP3, LC3II and Beclin-1 primary antibodies (1:2,000) at 4°C overnight. After washing with TBST 3 times, it was incubated again with secondary antibodies (1:10,000) at room temperature for 2 h. Finally, the color was developed using DAB away from light, and the protein gray level was recorded and photographed using a Bio-Rad gel imager. With GAPDH as a control, the protein in each group was quantitatively analyzed.

### Statistical analysis

SPSS22.0 software (IBM Corp., Armonk, NY, USA) was used for statistical analysis. Measurement data were expressed as (x ± s), and subjected to the independent t-test. Multigroup comparisons were conducted by one-way analysis of variance. P<0.05 was considered to be statistically significant.

## Results

### NDS, volume of cerebral infarction and brain water content

**Figure 1:**
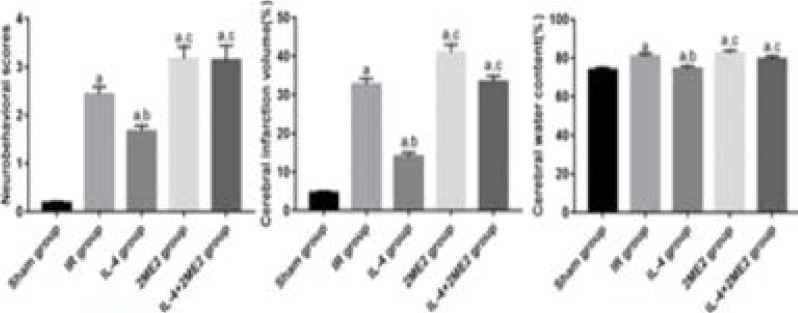
NDS, volume of cerebral infarction and brain water content (n=5 for each group, χ̅ ± s). ^a^P<0.05 *vs*. Sham group, ^b^P<0.05 *vs*. IR group, ^c^P<0.05 *vs*. IL-4 group.

The NDS, volume of cerebral infarction and brain water content were significantly increased in IR group compared with those in Sham group (P<0.05), they were decreased in IL-4 group compared with those in IR group (P<0.05), and they were incrased in 2ME2 group and IL-4+2ME2 group compared with those in IL-4 group (P<0.05) (Figure 1).

### Apoptosis and levels of SOD, MDA and ROs

**Figure 2:**
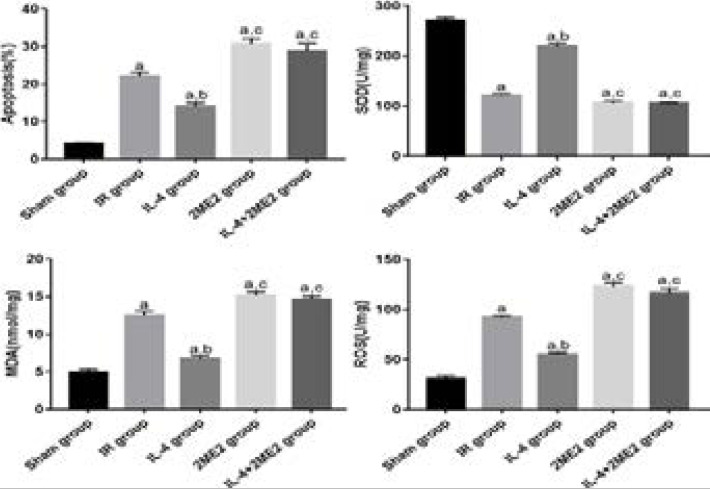
Apoptosis and levels of SOD, MDA and ROS (n=5 for each group, χ̅ ± s). ^a^P<0.05 *vs*. Sham group, ^b^P<0.05 *vs*. IR group, ^c^P<0.05 *vs*. IL-4 group.

IR group had significantly increased apoptosis and levels of MDA and ROS, but a significantly decreased level of SOD compared with Sham group (P<0.05). IL-4 group had decreased apoptosis and levels of MDA and ROS, but an increased level of SOD compared with IR group (P<0.05). The apoptosis and levels of MDA and ROS were increased, while the level of SOD was decreased in 2ME2 group and IL-4+2ME2 group compared with those in IL-4 group (P<0.05) (Figure 2).

### Levels of HIF-1α, BNIP3, LC3II and Beclin-1

**Figure 3:**
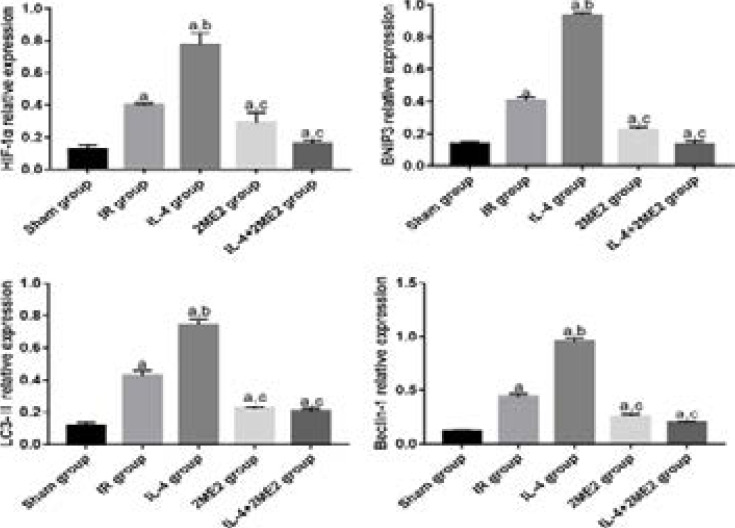
Levels of HIF-1α, BNIP3, LC3II and Beclin-1 (n=5 for each group, χ̅ ± s). ^a^P<0.05 *vs*. Sham group, ^b^P<0.05 *vs*. IR group, ^c^P<0.05 *vs*. IL-4 group.

The levels of HIF-1α, BNIP3, LC3II and Beclin-1 were significantly higher in IR group than those in Sham group (P<0.05), they were also significantly higher in IL-4 group than those in IR group (P<0.05), and they were significantly lower in 2ME2 group and IL-4+2ME2 group than those in IL-4 group (P<0.05) (Figure 3).

## Discussion

Due to ischemia and hypoxia in brain tissues, energy metabolism disorders are caused in the brain, so that ROS and excitatory amino acids are massively accumulated and released, resulting in autophagic flux. Beclin-1 and LC3 proteins have close associations with the occurrence of autophagy. Beclin-1, a homolog of yeast ATG6, can induce the positioning of other autophagy-related proteins in autophagic vacuoles, which is an important molecule during the formation of autophagosomes. LC3 is an autophagosomal membrane protein involved in all stages of autophagy, and it can serve as a marker for autophagy activity. After autophagy occurs, LC3-I is converted into LC3-II through ubiquitin modification, thereby initiating autophagy. He et al.^11^ showed that resveratrol could increase the LC3II/LC3I ratio, lower the expressions of p62 and NLRP3, and alleviate inflammatory injury caused by brain IR through enhancing autophagy activity, and that the expression of NLRP3 was enhanced by 3-MA pretreatment. Sun et al.^12^ found that MCAO indced autophagy that was further promoted after administration with eugenol, thereby preventing in vitro OGD/R injury and CIRI. Zhang et al.^13^ found that inducing chloride channel-3 (ClC-3) could raise the expression of Beclin-1 and activate autophagy, exerting a protective effect against CIRI, while knockdown of ClC-3 significantly worsened CIRI by inhibiting autophagy *in vivo*.

As a key factor in hypoxic response, HIF-1α is implicated in regulating mitochondrial autophagy, so that the release of ROS and pro-apoptotic factors is weakened, preventing further cell damage and improving cell survival. HIF-1α is an upstream gene of BNIP3, and activated HIF-1α enhances mitochondrial autophagy through up-regulating BNIP3, thereby exerting a cytoprotective effect. It has been confirmed that the HIF-1α/BNIP3 signaling pathway protects against MIRI by enhancing autophagy. For example, Liu et al.^14^ reported that Panax Notoginseng Saponins enhanced mitochondrial autophagy through activating the HIF-1α/BNIP3 signaling pathway in the rat model of myocardial injury, thereby exerting a significant protective effect against MIRI. Yang et al.^15^ found that deferoxamine combined with sevoflurane, through restoring and promoting HIF-1α/BNIP3-mediated mitochondrial autophagy, could alleviate MIRI in diabetic rats. Zhu et al.^16^ confirmed that berberine prevented MIRI by inducing myocardial cell proliferation, inhibiting myocardial apoptosis, and inducing HIF-1α/BNIP3-mediated mitochondrial autophagy. In the present study, IL-4 group had higher levels of HIF-1α and BNIP3 than IR group. After 2ME2 treatment, the levels of HIF-1α and BNIP3 declined in 2ME2 group and IL-4+2ME2 group, suggesting that IL-4 exerts a protective effect on the brain by activating the HIF-1α/BNIP3 signaling pathway.

## Conclusion

IL-4 can reduce apoptosis and oxidative stress through activating the HIF-1α/BNIP3 signaling pathway, thereby alleviating CIRI in mice. Regardless, this study is still limited. We only explored the role of IL-4 in reliving CIRI in mice based on the HIF-1α/BNIP3 signaling pathway, and whether there are other related pathways needs further study.
